# Progress of the Art of Macrophage Polarization and Different Subtypes in Mycobacterial Infection

**DOI:** 10.3389/fimmu.2021.752657

**Published:** 2021-11-09

**Authors:** Gai Ge, Haiqin Jiang, Jingshu Xiong, Wenyue Zhang, Ying Shi, Chenyue Tao, Hongsheng Wang

**Affiliations:** ^1^ Institute of Dermatology, Chinese Academy of Medical Sciences and Peking Union Medical College, Nanjing, China; ^2^ Imperial College London, London, United Kingdom; ^3^ National Center for Sexually Transmitted Disease and Leprosy Control, China Centers for Disease Control and Prevention, Nanjing, China; ^4^ Centre for Global Health, School of Public Health, Nanjing Medical University, Nanjing, China

**Keywords:** mycobacteriosis, granuloma, macrophages, multinucleated giant cells, epithelioid cells, foam cells

## Abstract

Mycobacteriosis, mostly resulting from *Mycobacterium tuberculosis* (MTb), nontuberculous mycobacteria (NTM), and *Mycobacterium leprae* (*M. leprae*), is the long-standing granulomatous disease that ravages several organs including skin, lung, and peripheral nerves, and it has a spectrum of clinical-pathologic features based on the interaction of bacilli and host immune response. Histiocytes in infectious granulomas mainly consist of infected and uninfected macrophages (Mφs), multinucleated giant cells (MGCs), epithelioid cells (ECs), and foam cells (FCs), which are commonly discovered in lesions in patients with mycobacteriosis. Granuloma Mφ polarization or reprogramming is the crucial appearance of the host immune response to pathogen aggression, which gets a command of endocellular microbe persistence. Herein, we recapitulate the current gaps and challenges during Mφ polarization and the different subpopulations of mycobacteriosis.

## Introduction

Mycobacteriosis is a contagious disease ravaging the skin tissue, respiratory system, and peripheral nerves, which results from *Mycobacterium tuberculosis* (MTb), nontuberculous mycobacteria (NTM), and *Mycobacterium leprae* (*M. leprae*). Tuberculosis (TB), caused by the MTb complex, has plagued humanity when it has killed billions of populations over the past two centuries (1). Cutaneous TB, including 1% to 2% of all cases, is a rare clinical manifestation of MTb or *M. bovis* infection. Rapidly growing mycobacteria, such as *M. abscessus* group, *M. fortuitum* group, *M. mucogenicum*, and *M. smegmatis* and slow-growing mycobacteria, such as *M. avium* complex, *M. kansasii*, and *M. marinum*, are compose of NTM (2). NTM cutaneous infection is unwonted, and predisposing factors, such as skin injury (such as gardening and fish-related injuries, injections, and surgery) or immunosuppression make up 95% of cases (3). Leprosy, Hansen’s disease, is a remarkable public health problem, especially in countries such as Brazil, India, and Indonesia (4). Leprosy is a neglected tropical disease encountered by *M. leprae* or *M. lepromatosis*. At present, effective vaccines against infection and markers for beneficial immunity are not available (5, 6). The inability to eradicate the bacteria can result in infection in the immune system in a granuloma structure. Macrophages (Mφs), primary effectors of inherited response, are considered essential pathophysiologic factors in wide-spread disease procedures involved with chronic inflammation. The heterogeneity of Mφs, either due to their developmental origin or their particular activation morphologies, is becoming increasingly distinct with regard to their diverse roles within infection of microbes (7). As a central part of the innate immunity and as the paramount host of infectious granuloma pathogens, Mφs have been the central focus of mycobacteriosis investigation.

## Infectious Granuloma

**Figure 1:**
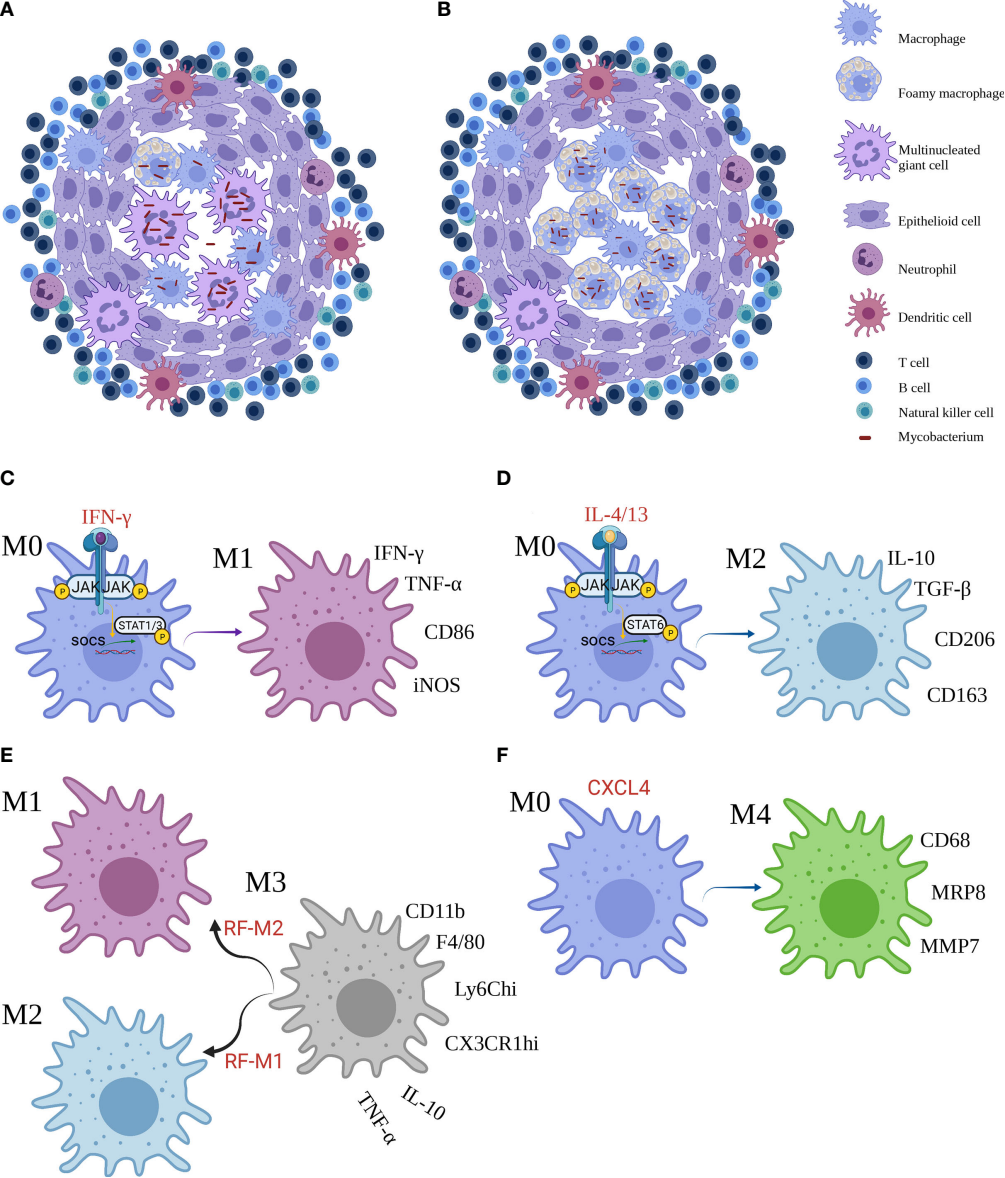
The granuloma and macrophages subsets. **(A, B)** Two frameworks of mycobacterial infection granuloma. **(C–F)** Reprogramming of macrophages and main markers of M1, M2, M3 and M4 Mϕ. Created with BioRender.com.

Granuloma is a highly structured and organized collection of Mφs, often with phenotypic switches and other immune cells recruited, including multinucleated giant cells (MGCs), epithelioid cells (ECs), and foam cells (FCs). Someone claimed a new *ex vivo* granuloma culture technique to study granuloma consolidation (8). Mechanistically, Cronan et al. have found that in the existence of robust interferon-gamma (IFN-γ) signaling immune response, confronting interleukin (IL)-4 and IL-13 signals were associated with Mφ epithelial transition. IL-4/13 signaling, induced by *stat6*, was required for epithelioid transformation and granuloma architecture. Apart from *stat*6 function required in the new granuloma formation, persistent *stat*6 pathway was required to maintain the expression of E-cadherin and granuloma (9). MAB_4780, encoding a dehydratase, was required for intracellular *M. abscessus* growth and to avoid lysosome-mediated degradation, which compromises survival of ΔMAB_4780 in Mφs and granuloma formation (10). In granuloma transformation, IFN-γ and tumor necrosis factor-alpha (TNF-α) were deemed to be effective regulators, whereas IL-10 was a passive effector. Intriguingly, etanercept and adalimumab, the human monoclonal anti-TNF-α IgG1, exacerbated M1 polarization and delayed MGC generation in granuloma (11). Magically, there are two types of granulomas in leprosy. At one pole of leprosy, the presence of MGCs and granuloma configuration in tuberculoid leprosy (TT) contributes to the containment of *M. leprae* proliferation and transmission (**Figure 1A**). At the other pole, lepromatous leprosy (LL) has phagocytic FCs heavily parasitized with freely multiplying intracellular *M. leprae* (**Figure 1B**) (12). Ma et al. have constructed a map *via* integrating single-cell RNA sequencing with spatial sequencing to identify that the primary cell types, consisting of T cells, Mφs, keratinocytes, endothelial cells, and fibroblasts, were described to research the cellular composition and status discrepancies between reversal reactions and LL, and LGCs are more frequent in both lesions. IL-1β and IFN-γ were supposed to be important upstream effectors of the pseudo time trajectory and the activation of Mφs in granulomas to product genes contributing to antimicrobial responses in human leprosy granulomas (13).

Granuloma is a leading gateway for the host immune response to microorganisms and shape immune interplays, disease progression, and degeneration (14). The granuloma is a functional paradox, for example, it contains the bacilli in a local reservoir, preventing mycobacterial dissemination to near normal tissues of the host, or to shield itself from host’s immunologic killing mechanisms and antimicrobial response (15).

## Mφ Phenotypes in Mycobacteriosis

Mφs show the increment of plasticity, and they can be differentiated into two contrary subsets: M1 Mφs and M2 Mφs. The network of molecular mediators is regulated in response to the diversity of stimulus. Binding of IFN-γ to its cellular surface receptor, for example, induces the activation of receptor-linked JAKs, which trigger STAT1 to dimerize and translocate to the nucleus where it initiates gene transcription that skew toward M1-correlated functions such as promoted microbicidal activity and proinflammatory cytokine production (**Figure 1C**). By contrast, IL-4 and IL-13 activate STAT6 to promote M2 profile inhibiting these effects (**Figure 1D**) (16). Furthermore, Yun−Ji et al. have shown that c-JUN N-terminal kinase (JNK)-mediated M1 plasticity was important in the elimination of bacilli *via* p53-mediated apoptosis by Mφs. Similarly, virulent MTb H37Rv infection can induce M2 Mφs and in turn restrain p53 by the activation of mouse double minute 2 (MDM2). These consequences elucidated that M2 population decreases p53-elicited cell death by MDM2 induction (17). In addition, early secreted antigenic target of 6 kDa (ESAT6), a prerequisite step to support the formation of granuloma, was one of the effectors used by MTb to facilitate the proinflammatory M1 Mφ at the primo-infection and then triggered the switch of M1 to M2 Mφ at a later infection phase (18). In particular, proinflammatory environment or bacterial product could perturb the classical M1 or M2 phenotypes. Bénard et al. recently showed that type I IFN hyperproduction by MTb-stimulated B cells drove an altered Mφs polarization toward a regulatory/anti-inflammatory profile, namely, M2 Mφ, during TB which associated with increased MTb burden in lungs (19). Moreover, Mφ polarization may augment antimicrobial response against MTb in the existence of vitamin D (20).

Furthermore, high-mobility group N2 (HMGN2) regulates anti-NTM-inherited response function of Mφ. In addition, HMGN2 is triggered in NTM and IFN-γ-primed M1-skewed subpopulation polarization (21). Yet, *M. abscessus* infection robustly induced p38 MAPK-dependent heme-oxygenase-1 (HO-1) induction in the THP-1 cells. HO-1 production was important for *M. abscessus* growth during the early stages of infection, and that the HO-1 producted bilirubin and biliverdin, perhaps through modulation of intracellular ROS levels, may be involved (22). Glycopeptidolipids limited the virulence of *M. abscessus* among Mφs by inhibition of apoptosis and spreading of bacteria (23). In TT, the activation of the classical signal by M1 Mφs results in the expression of TNF-α, IFN-γ, and iNOS, which trigger the multiplication of free radicals that remove *Bacillus* (24). Moreover, the LL shows a superiority of M2 Mφs that promotes the expression of IL-10, transforming growth factor-β, fibroblast growth factor-β, Arg-1, CD206, and CD163, causing immunosuppressive response and tissue repairment (25, 26). *M. leprae* could then utilize infected Mφs by two mechanisms: first, *M. leprae*-infected Mφs preferentially activated Treg but not Th1 or cytotoxic T-cell responses; second, *M. leprae*-infected Mφs were effective in escaping CD8^+^ T-cell-primed cytotoxicity (27).

Other than the M1 and M2 subpopulations, a M3 switch profile exists. The M3 Mφ could be divided into two subsets such as the M1/2 paradigm, which in response to a reprogramming factor M1 (RF-M1) skews toward M2 Mφ, and the M2/1 dichotomy, which responding to RF-M2 favors M1 Mφ (28). In murine mesothelioma microenvironment, flow cytometry disclosed that the mixture of M1 and M2 phenotypes (CD11b^+^ F4/80^+^ Ly6C^hi^ CX3CR1^hi^), that was, M3 Mφ, secreted IL-10 and TNF-α. Jackaman et al. have suggested that the shifts of M1 to M2 Mφ and vice versa could occur through the M3 changing formation (**Figure 1E**) (29). The M3 mediator can be triggered by upregulation of M1-reprogramming signals with coinstantaneous suppression of the M2 Mφ transcription factors, STAT3, STAT6, and/or SMAD3 in Ehrlich ascites carcinoma (30). Nevertheless, the role of M3 Mφ in mycobacteriosis remains undetailed, and more studies are required for further investigation.

Unluckily, the part of M4 macrophages following M3 macrophages in Mφ phenotypes in mycobacteriosis notably, considerable evidence for another subpopulation of Mϕ, namely, M4 Mϕ, was frequently observed. In the presence of CXCL4, M0 Mϕ changed to M4 Mϕ, expressing CD206, CD68, matrix metallo proteinase (MMP) 7, myeloid-related protein 8 (MRP8) and S100A8, producing IL-6, TNF-α, MMP7, and MMP12 in atherosclerosis and cardiac remodeling (31–33). At date, de Sousa et al. have also characterized the existence of M4 Mϕ in leprosy. Immunostaining determined that the expression of CD68, MRP8 and MMP7 was significantly higher, while IL-6 and TNF-α was significantly lower in the LL group compared with the TT group. The higher expression of M4 profile in LL lesions implied that the subpopulation was ineffective in the removal of bacilli, resulting in the development of multibacillary form and microbes replication (**Figure 1F**) (34). Further work is necessary to robustly establish this mechanism. Notwithstanding, the role of the new subset in TB and NTM is unclear.

## MGCs in Mycobacteriosis

Specific lineage of MΦs, particularly MGCs containing a horseshoe-shaped ring of nuclei, contributes to the core of granulomas. Previously, cells with three nuclei and the expression of iNOS were markers for MGC transformation (35). In addition, the formation of MGC, involving cell fusion (36), was a Mφ-specific, evolutionarily ancient program that proceeds in response to the persistence of extrinsic and intrinsic stimuli (37). Mφs or monocytes can be transformed into MGCs under several statuses, including cultivating with IL-4 or IL-13, GM-CSF combined with IL-4, IFN-γ bounding with IL-3, or bacterial glycolipids. E-cadherin is a necessary player in fusion, and its production can be stimulated by the activation of STAT6 through IL-4 or IL-13 pathway, similar to epithelialization under the circumstance of schistosome granulomas (38). However, the development of polyploid MGCs involves cell autonomous affliction of Toll-like receptor-elicited DNA damage, cell autonomous cell-cycle alterations, and impairment of p53 function by the potent antimicrobial effector, namely, NO, driving mitotic defects and multinucleation (35, 39). Wang et al. have corroborated experimental evidence that IL-15 primes M1 Mφ transformation, reprograms peripheral blood mononuclear cells in humans to transform into MGCs *via* direct activation of T cells and myeloid cells (40). Queval et al. have shown that out of the four infection combinations (blood-derived primary human and bovine Mϕs [hMϕ or bMϕ, respectively] infected with *M. bovis* and MTb), bMϕ infected with *M. bovis* promotes the formation of MGCs. Mechanistically, they have distinguished the functional differences between *M. bovis* and MTb host-pathogen interplay and demonstrated that MPB70 from *M. bovis* and extracellular vesicles released by *M. bovis*-infected bMϕ promote Mϕ multinucleation (41). Startlingly, local adaptive immune response, particularly programmed cell death ligand-1, fatty acid, and cholesterol metabolism could take part in containing granuloma progression in human lung TB (42, 43).

Unfortunately, the distinct role of MGCs in mycobacterial infection immune response remains as major gaps. MGCs may restrict mycobacterial cell-to-cell dissemination, involve in mycobacterial latency, or promote tissue destruction because of their high expression of extracellular matrix-degrading epithelioid macrophage marker molecules (EMMMs) (38, 43). The maturation of MGCs supplies a restrictive environment for *M. bovis*. The major lysosomal degradative signals remain functional within MGC transition. In addition, the increase of *M. bovis* in acidified compartments and correlation with LC3B in matured MGCs indicates that MGCs presented a restrictive milieu for microorganism replication (41). Nonetheless, the role of MGCs in NTM and leprosy remains an elusive issue.

## ECs in Mycobacteriosis

Microscopic analysis discloses that tightly interdigitated cell membranes are formed in zipper-like arrays to resemble epithelioid histiocytes. Nevertheless, none of the fusion molecules is strictly required to give rise to ECs, and the procedure is complicated. Epithelial differentiation can occur during days of granuloma transformation. Using the *M. marinum*-zebrafish model, Cronan et al. have found recently that granuloma Mφs undergo reprograming, which involves E-cadherin-dependent formation of fusogenic epithelial cell (44). In TB, ESAT6 plus TLR2 can activate iNOS/NO and ROS signaling to reduce the trimethylation of H3K27, thereby promoting the expression of EMMM that improved the transformation of Mφs into ECs (45).

The EC functions are amphibolous and nebulous from being repleted with organelles and strongly phagocytic and microbicidal to being nonphagocytic cells with secretory functions, which might be adjunctive in granuloma function. Notwithstanding, some people have demonstrated by electron microscopy that the ECs in TB are “primarily biosynthetic rather than phagocytic” (46). However, ECs control the multiplication of mycobacteria at least in one experimental model. Previous dates, therefore, have elucidated that interference to E-cadherin production, a tight junction protein among ECs, enhanced the transformation of untightly structured granuloma, resulting in unrestricted MTb motion and leads to MTb regeneration and dissemination (47). In NTM, EC surrogates restrain *M. avium* growth and serves as APCs *in vitro* and *in vivo*. ECs were commonly seen in TT and borderline tuberculoid leprosy (BT). Inconceivably, ECs from TT granulomas exhibited the M1 phenotype (CD68^+^ CD163^−^), whereas Mφs in LL granulomas showed the M2 phenotype (CD68^+^ CD163^+^) (48).

## FCs in Mycobacteriosis

FCs, with deregulated lipid metabolism, are a manifestation of maladaptive responses in chronic inflammatory statuses (49, 50). The biogenesis of FCs varies with underlying diseases. FC biogenesis is involved in the disruption of cholesterol homeostasis and consequent endocellular accumulation of cholesteryl esters in atherosclerosis, but it is linked to triglyceride accumulation in hMφs infected with MTb that is elicited by TNF receptor pathway *via* downstream activation of the caspase cascade and mammalian target of rapamycin complex 1 (51). In comparison, Genoula et al. suggested that alternatively activated Mφs were loath to the accumulation of lipid droplets (LDs) *via* the STAT6, which facilitated the degradation of lipids. However, MTb offsets lipolysis *via* switching alternatively activated Mφ metabolism to accumulate LDs due to the HIF-1α activation (52). Similarly, the zebrafish-*M. marinum* granuloma contained FCs and the mycobacterial ESX1 pathogenicity locus thought to elicit the morphology switch of Mφs to FCs (53). The biogenesis of FCs in leprosy remains a challenging enigma.

Diverse, and in part controversial, we summarize the current findings in the role of FCs in mycobacteriosis. First, Mφ ontology may be a major paramount factor of the inherited response in the containment of MTb infection. LDs may take part in inherent immunity against MTb by directly eliminating intracellular MTb and modulate metabolism to infection (54). Strikingly, PPAR signaling is responsible for lots of adipocyte differentiation-correlated genes, leading to amassing of intracellular lipids to accommodate *M. leprae* parasitization in host FCs (55, 56). Furthermore, the formation of LD may support the host by averting access of MTb to host’s fatty acids (FAs) while favoring native immune responses (54). In comparison, unlike other programs, FC formation reduced the avidity of host cell and the phagocytosis of MTb while protecting the cells from death. The protective effect is associated with enhanced inflammatory potential of FCs and cause slower proliferation of MTb. Also, the balance of TNF-a, IL-1β, IL-6, and NF-kB innate inflammatory responses was altered in response to MTb vs. LPS in FCs compared with uninfected controls (57). Additionally, FCs triggered the formation of necrotic core by releasing triglyceride-rich content into the caseum (51), resulting in progressive lung tissue destruction and pulmonary function loss in infected rabbits and marmosets and in individuals with active TB (50).

Lastly, FCs may result in TB pathogenesis by enhancing MTb persistence and drug resistance. Moreover, a lipid-rich diet rather than nutrient deprivation in caseum rewires the condition of MTb toward drug resistance (58). In addition, IL-10/STAT3 axis primed FC differentiation during MTb infection, favoring pathogen persistence (59). Palma et al. have shown that controlled caloric restriction protected murine model against pulmonary MTb infection by decreasing bacterial load and FC proliferation to reduce lung damage and limit MTb spreading (60). Thus, the reduction of LDs in MTb-infected FCs might restrain the endocellular survival of MTb (61). Likewise, ultrastructural analysis of demic leprosy tissue showed colocalization between cholesterol-laden lipid bodies and *M. leprae*-containing phagosomes in FCs. The mechanisms of leprosy indicate that lipid abundance has a pathophysiological effect on the persistence of microbes in the host. The function of FCs remains the unsolved mystery of NTM.

## Discussion

*Mycobacterium*-infected disease is an infectious granuloma disease with a spectrum of clinical and pathological features. Granuloma formation and immune mechanism are primarily observed in mycobacteriosis. Different cellular immune and clinical manifestations are primed by Mφ polarization or reprogramming. Different Mφ subphenotypes may be positively correlated with the number of germs and host immune response. The increment of M2 Mϕs and FCs and a low degree of MGCs are more likely to attribute to the bacillary multiplies and impaired innate immune. Conversely, the results reveal a positive correlation between high-level M1 Mφs and MGCs, the diminution of FCs, and a limited bacterial load and immunocompetent innate immune response. Particularly, ECs are commonly seen in TT and borderline BT, FCs are mostly a commonly factor in leprosy, particularly LL. Now, we recapitulate the main findings of Mφs, MGCs, ECs, and FCs in mycobacterial infection (**Table 1**). Mφ reprogramming or markers can shed light on the cell immune response in mycobacteriosis. Moreover, the mycobacterial granuloma model may delineate the development of alternative vaccines for mycobacteriosis. Accordingly, these researches prompt that Mφs, especially M1 Mφ and LGCs represent a therapeutic target for the emergence of antibacterial immunity. Together, therapies targeting some particular cells are being studied as novel therapies for TB, leprosy, and other bacterial infections.

**Table 1:** Main findings described in Mφs, MGCs, ECs, and FCs.

Cell types	Stimulus	Main cyto/chemokines and enzymes	Functions/Immune responses	References
M1 Mφ	IFN-γ/STAT1, p53, ESAT6	iNOS, IFN-γ, TNF-α, CD86, IL-6, and HMGN2	Microbicidal activity and proinflammatory cytokine production	(16–18, 24)
M2 Mφ	IL-4 plus IL-13/STAT6, ESAT6, type I IFN	Arg-1, IL-10, TGF-β, fibroblast growth factor-β, CD206, CD163	Immunosuppressive response and tissue repairment	(16–19, 25, 26)
M3 Mφ	RF-M1/2	IL-10, TNF-α, CD11b, F4/80, Ly6C^hi^ CX3CR1^hi^	Undetailed	(28, 29)
M4 Mφ	CXCL4	CD68, MRP8, MMP7	Weak phagocytosis, favoring bacillus regeneration	(35–38)
MGC	IL-4 or IL-13, GM-CSF plus IL-4, IFN-γ plus IL-3, E-cadherin, IL-15	iNOS, EMMMs, PD-L1	Inhibiting mycobacterial cell-to-cell spread or tissue destruction and mycobacterial latency	(39, 42, 45–47)
EC	ESAT6 plus TLR2	CD68^+^ CD163^-^, CD68+ CD163+	Strongly phagocytic and microbicidal or nonphagocytic cells with secretory functions	(49, 52)
FC	PPAR, IL-10	TNF-a, IL-1β, IL-6	Favoring inherited response or pathogen persistence, Less-bactericidal, Less-phagocytic	(59–61)

Arg-1, arginase-1; CXCL, C-X-C motif ligand; ECs, epithelioid cells; EMMMs, extracellular matrix-degrading epithelioid macrophage marker molecules; ESAT6, early secreted antigenic target of 6-kDa; FCs, foamy cells; HMGN2, high-mobility group N2; IFN-γ, interferon-gamma; IL, interleukin; iNOS, inducible nitric oxide synthase; IRF, Interferon regulatory factors; Mφs, macrophages; MGCs, multinucleated giant cells; MMP, matrix metallo proteinase; MRP8, myeloid-related protein 8; PD-L1, programmed cell death ligand-1; RF-M1, reprogramming factor M1; STAT, signal transducer and activator of transcription; TGF-β, transforming growth factor beta; TNF-α, tumor necrosis factor-alpha.

## Author Contributions

HW, HJ, JX, WZ, and YS involved in supervision. CT and GG drafted figures. GG reviewed the literature and wrote the manuscript. All authors contributed to the article and approved the submitted version.

## Funding

This study was supported by grants from the Science and Technology Planning Project of Jiangsu Province of China (BE2018619), Chinese Academy of Medical Science Innovation Fund for Medical Science (2017-I2M-B&R-14), and the National Natural Science Foundation of China (81972950).

## Conflict of Interest

The authors declare that the research was conducted in the absence of any commercial or financial relationships that could be construed as a potential conflict of interest.

## Publisher’s Note

All claims expressed in this article are solely those of the authors and do not necessarily represent those of their affiliated organizations, or those of the publisher, the editors and the reviewers. Any product that may be evaluated in this article, or claim that may be made by its manufacturer, is not guaranteed or endorsed by the publisher.
